# Ultrasound-guided regional anesthesia simulation: use of meat glue in inexpensive and realistic nerve block models

**DOI:** 10.1186/s12909-019-1591-1

**Published:** 2019-05-15

**Authors:** Leily Naraghi, Judy Lin, Kay Odashima, Simran Buttar, Lawrence Haines, Eitan Dickman

**Affiliations:** Maimonides Medical Center, Department of Emergency Medicine, 4802 10th Ave, Brooklyn, NY 11219 USA

**Keywords:** Regional anesthesia, Simulation, Ultrasound, Point-of-care, Meat glue, Nerve block model

## Abstract

**Background:**

Ultrasound-guided regional anesthesia (UGRA) is increasingly used by emergency physicians to provide safe and effective pain relief for patients. However, one of the factors limiting its widespread use is the lack of realistic models available for learners to train on. There are currently no inexpensive nerve block models available that are injectable and that closely mimic nerves, fascial planes, muscles, and other landmarks. Our aim is to create inexpensive, injectable nerve block models that can be used as effective medical training tools for UGRA.

**Methods:**

By using a lean cut of pork such as pork loin, yarn soaked in ultrasound gel to simulate peripheral nerves, and drinking straws filled with gel to represent vascular structures, we created various nerve block models. Meat glue applied between sections of meat appears hyperechoic under ultrasound, thereby mimicking fascial planes and has the added benefit of helping to secure the components of the model together. Using these elements, we were able to create realistic peripheral nerve, fascia iliaca compartment, serratus anterior plane, and interscalene brachial plexus models.

**Results:**

One of the necessary skills in performing UGRA involves placing the needle tip along a fascial plane and visualizing hydrodissection of this plane with the local anesthetic. When meat glue (transglutaminase) is applied between layers of meat such as pork loin, the meat binds together and creates a hyperechoic line that mimics a fascial plane. When meat glue is applied to two apposing fascial layers naturally occurring on the meat, the fascial plane can be injected, and fluid can be seen hydrodissecting in this space. We created several nerve block models using meat glue and other components to mimic normal landmarks.

**Conclusions:**

We have developed inexpensive and easily reproducible models that create the realistic appearance of tissues, nerves, and fascial planes under ultrasound. They can also accurately simulate hydrodissection of fluid in fascial planes. We hope these nerve block models will allow for the education in UGRA to be more widespread and accessible to learners from all specialties.

**Electronic supplementary material:**

The online version of this article (10.1186/s12909-019-1591-1) contains supplementary material, which is available to authorized users.

## Background

Ultrasound Guided Regional Anesthesia (UGRA) is increasingly used by emergency physicians as a safe and effective method of pain control in patients with various musculoskeletal injuries [1–4]. Regional anesthesia has been shown to reduce the utilization of systemic analgesics and their subsequent side effects [1, 2, 5]. Examples of commonly used UGRA in the emergency department include fascia iliaca compartment blocks in patients with hip and proximal femur fractures [1, 2, 5–8], interscalene brachial plexus blocks in patients with shoulder dislocations [9], serratus anterior plane blocks in patients with rib fractures [10], and forearm nerve blocks in patients with hand injuries [10–13]. Studies have shown that medical training on simulation models is an effective way to learn new procedures and ultrasound techniques [14–16]. However, there are currently no inexpensive nerve block models available that are injectable and that closely mimic nerves, fascial planes, muscles, and other anatomical landmarks.

We used readily available and inexpensive material to create various training models that closely mimic the normal sonographic landmarks and on which needle insertion can be practiced under sonographic guidance. We also introduce the novel use of meat glue (transglutaminase) as a way to bind components of the model together and to create fascial planes that can be hydrodissected with fluid injected through a needle. These realistic models offer learners the ability to test and practice techniques that are not possible in other UGRA models. Two of these models, the serratus anterior plane and fascia iliaca compartment model were incorporated into a nerve block workshop for emergency medicine attending staff. After the workshop, attending physicians were surveyed regarding the fidelity and effectiveness of these models in their education.

## Methods

### Materials

- Approximately 2–3 lbs. of boneless pork tenderloin ($5.50/lb.)
  - Pork tenderloins was bought from local meat market. There was no permission necessary to buy and use the Pork tenderloins.
- Straws (regular $0.01ea, wide $0.07ea)
- Ultrasound gel ($4.00/bottle)
- Chewing gum ($1.50/pack)
- Meat glue ($12.00/6 oz. bag)
- Yarn ($5.00/roll)

All of our models are constructed using a combination of the following components:

1. Pork tenderloin: Meat is cut into shape of desired muscle groups. Meat with an overlying layer of fascia is preferred for optimal ability to hydrodissect along fascial planes.
2. Nerve: Yarn is soaked in ultrasound gel and any air trapped between the fibers is squeezed out. We find that using thicker “chunky” yarn yields better results. Several strands of yarn can be braided or twisted together to form thicker nerves.
3. Fascia: Meat glue is sprinkled between meat layers to simulate fascia and bind pieces of meat together.
4. Large Vessels: Large size straws are filled with ultrasound gel and occluded on both ends with already chewed chewing gum.
5. Interscalene Brachial Plexus: Regular thin size straws are filled with ultrasound gel and occluded on both ends with already chewed chewing gum to simulate the hypoechoic appearance of these nerves.

### UGRA model construction

#### Peripheral nerve model

**Fig. 1:**
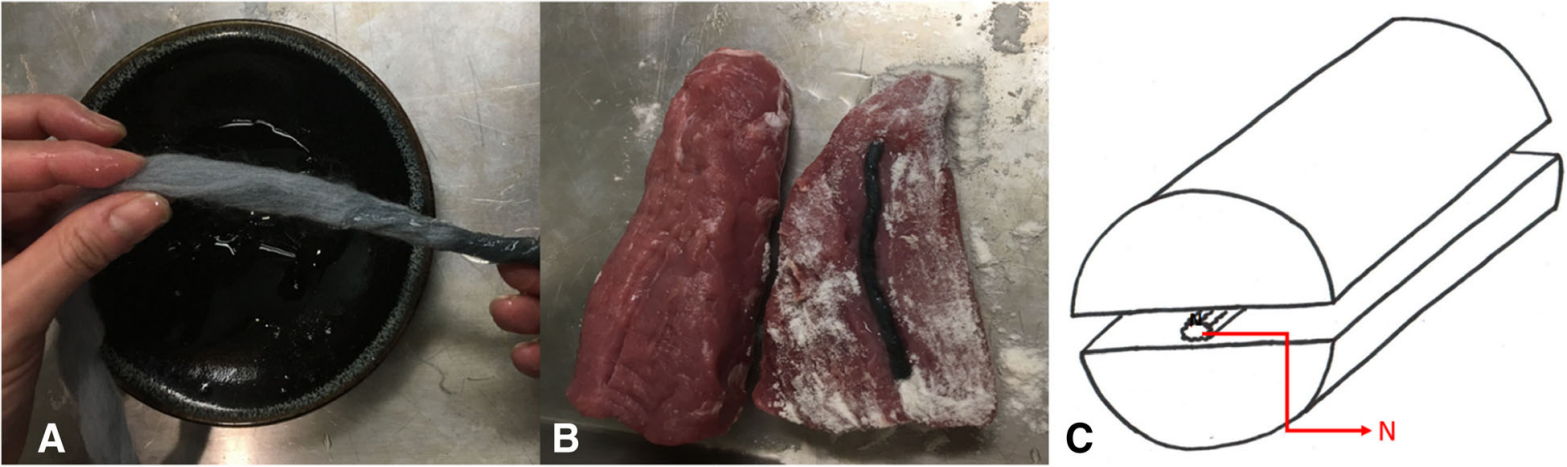
Gel soaked yarn (**a**). Yarn between the two meat layers sprinkled with meat glue (**b**). Cartoon representation of assembled model (**c**). N, nerve

Gel-soaked yarn is placed between two pieces of meat sprinkled with meat glue (Fig. 1a and b). A cartoon representation of this model is shown in Fig. 1c.

#### Fascia Iliaca compartment model

**Fig. 2:**
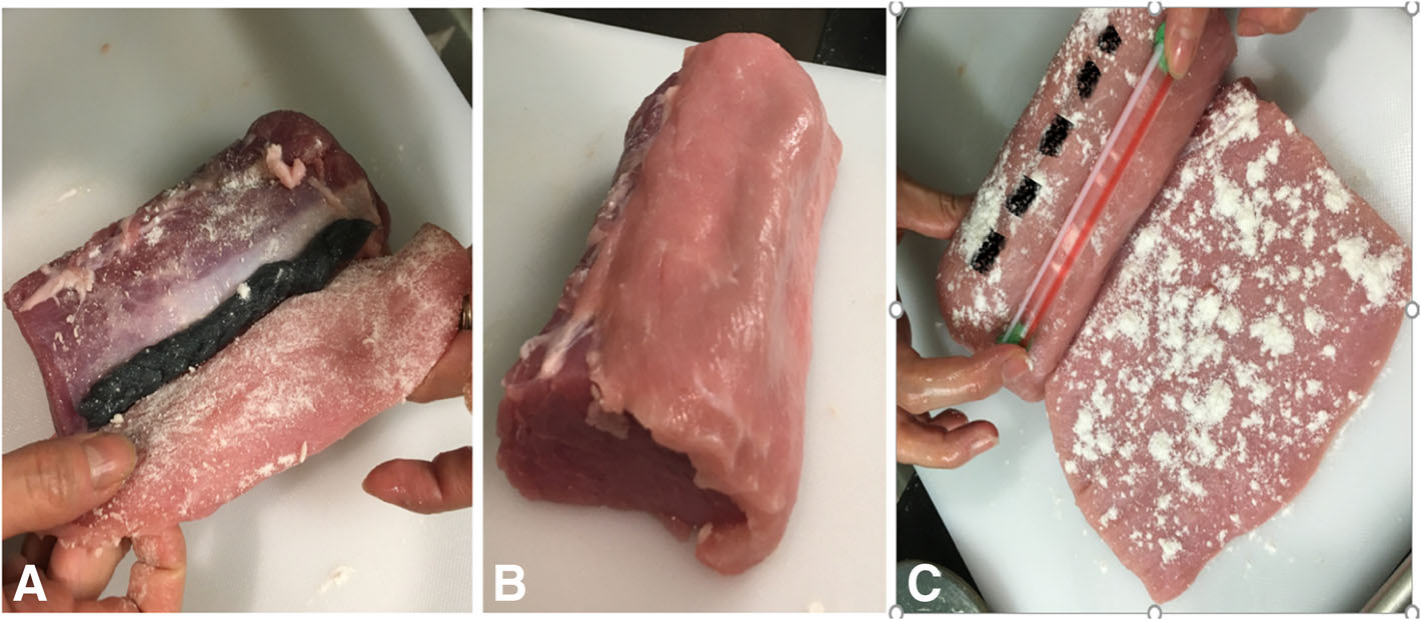
The yarn (femoral nerve) between the cylindrical piece of meat (iliopsoas), and the thin layer of meat (fascia iliaca) that is about to be placed on top (**a**). The thin slice of meat (fascia iliaca) covering the yarn (**b**). The gel filled straw (femoral artery) placed on top of the first thin slice of meat (fascia iliaca) lateral to where the yarn was placed underneath (broken line) (**c**)

**Fig. 3:**
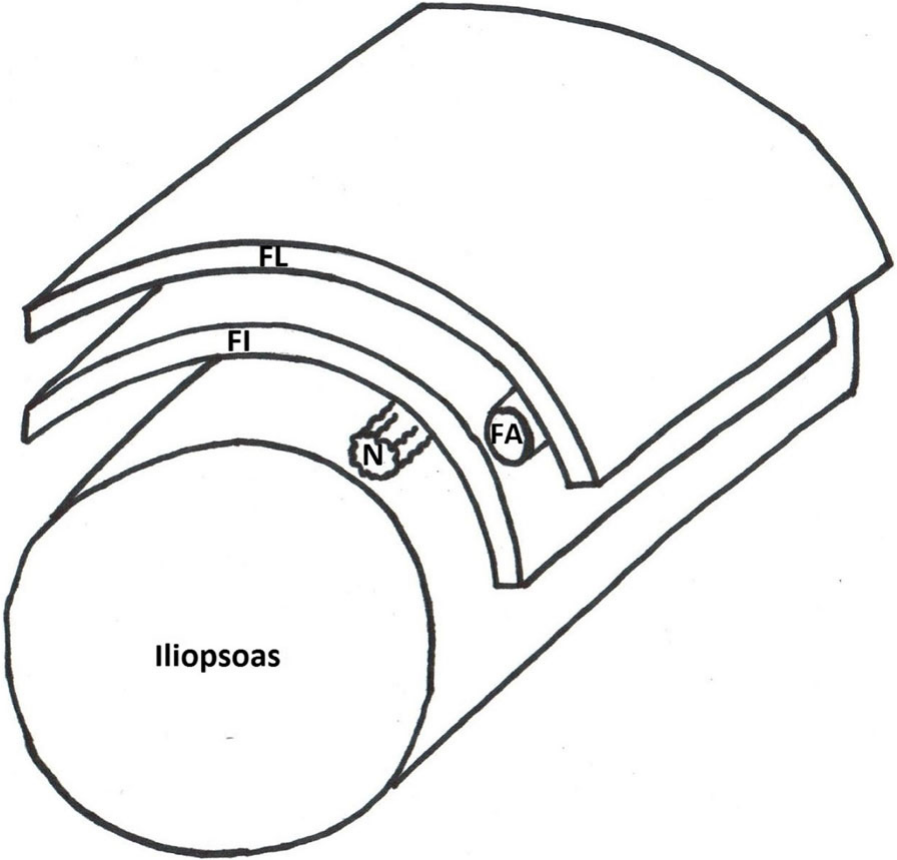
Cartoon image of the assembled fascia iliaca compartment model. N, nerve; FA, femoral artery; FL, fascia lata; FI, fascia iliaca

You will need three pieces of meat: one cylindrical piece representing the iliopsoas muscle, and two thinner slices which represent the fascia lata and the fascia iliaca respectively. Ideally, the meat layers will have naturally occurring fascia. The authors have found that by apposing naturally occurring fascia and securing them together with meat glue a realistic appearance of hydrodissection is achieved when the model is injected. For the fascia iliaca compartment model, the apposition of naturally occurring fascia should be between the cylindrical piece of meat and the layer of meat just above. Sprinkle meat glue over all surfaces of meat that are in contact with other surfaces of meat to ensure that these layers will bind together. Place the gel-soaked yarn representing the femoral nerve between the cylindrical piece of meat representing the iliopsoas and the thin slice of meat representing the fascia iliaca (Fig. 2a and b). Then, fill a wide straw with gel and occlude both ends with chewing gum. Place the wide gel filled straw on top of the slice representing the fascia iliaca lateral to where the yarn is below it, in order to represent the correct relationship of the femoral artery to the femoral nerve. Finally, place the second thin slice of meat representing the fascia lata on top, covering the straw (Fig. 2c). A cartoon representation of this model is shown in Fig. 3.

#### Interscalene brachial plexus model

**Fig. 4:**
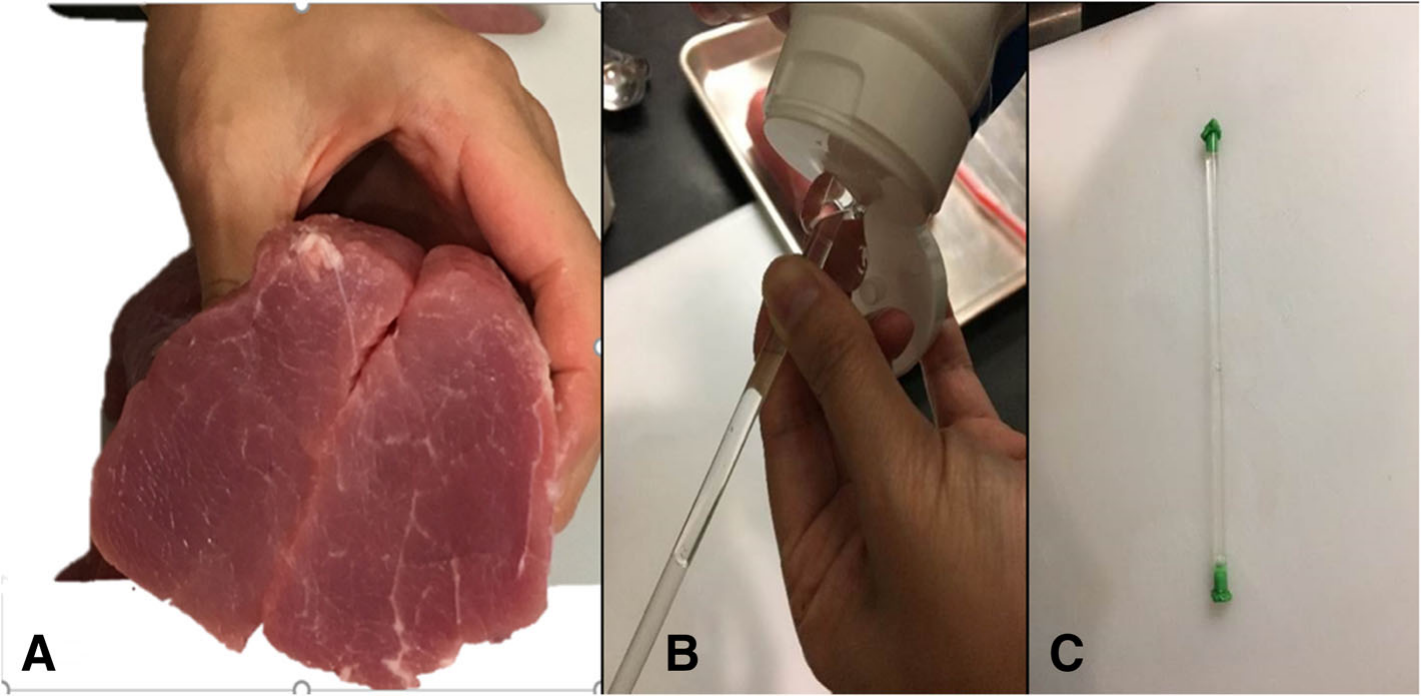
Two halves of a cylindrical piece of meat representing the anterior and middle scalene muscles (**a**). Gel-filled straw with chewing gum occluding the ends (**b** and **c**)

**Fig. 5:**
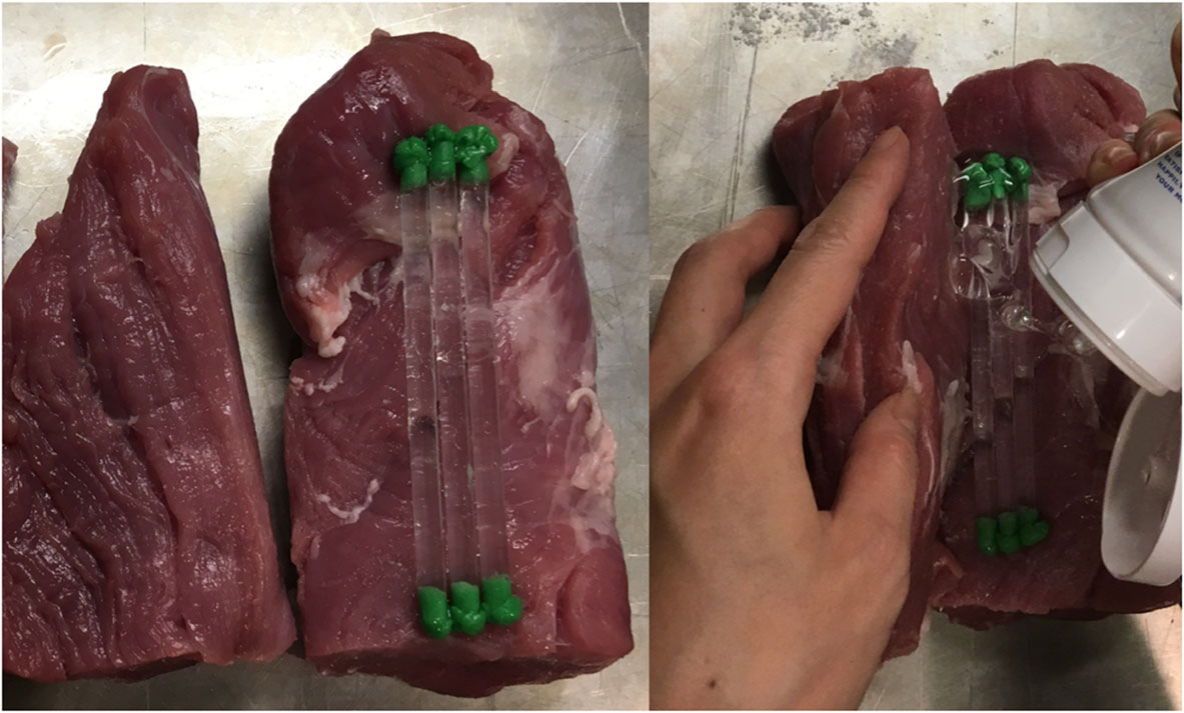
Three gel filled straws representing the interscalene nerve trunks with gel in between them

Cut a wedge-like slice of meat that is approximately 1 in. thick on one end. This represents the sternocleidomastoid muscle. Then, cut a cylindrical piece of meat lengthwise in half. The two halves represent the anterior and middle scalene muscles (Fig. 4a). Fill three regular straws with ultrasound gel and occlude the ends with chewing gum (Fig. 4b and c). These will represent nerve roots C5, C6 and C7 of the interscalene brachial plexus. Sprinkle the inner surfaces of meat with meat glue and place the straws side by side on one of the pieces. Spread a thin layer of ultrasound gel around the straws to eliminate any air pockets prior to opposing the two halves of the pork loin (Fig. 5).

**Fig. 6:**
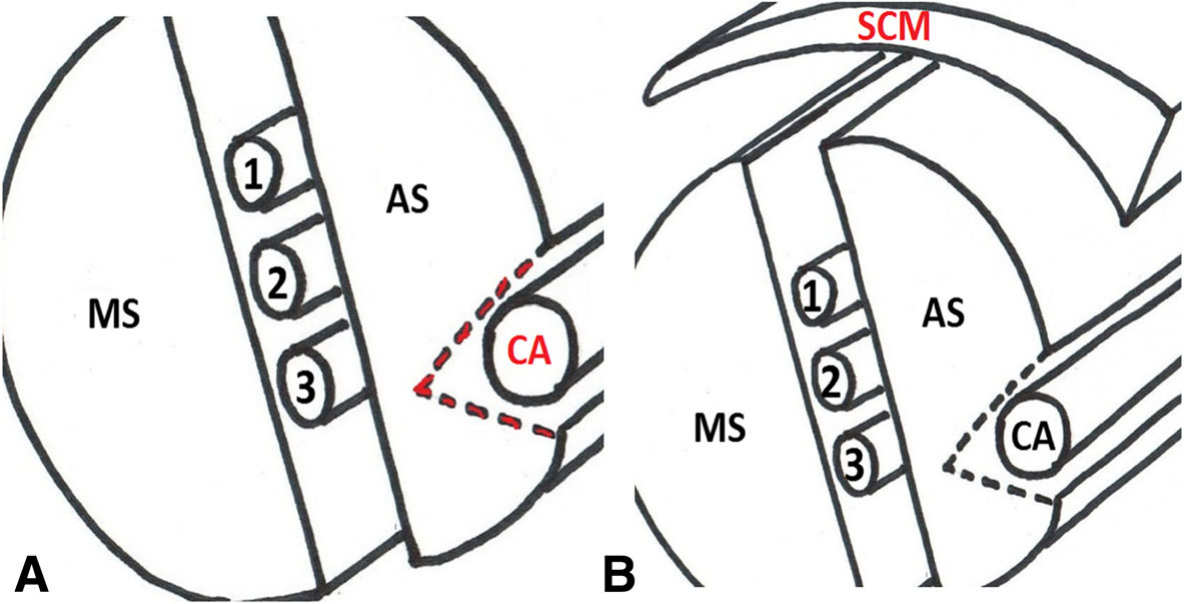
Incision in red indicates the location of the carotid artery (**a**). Thin layer of meat covering the model simulates the sternocleidomastoid muscle (**b**). SCM, sternocleidomastoid muscle; AS, anterior scalene muscle; MS, middle scalene muscle; CA, carotid artery; the interscalene brachial plexus is located between AS and MS muscles; 1, C5 nerve root; 2, C6 nerve root; 3, C7 nerve root

**Fig. 7:**
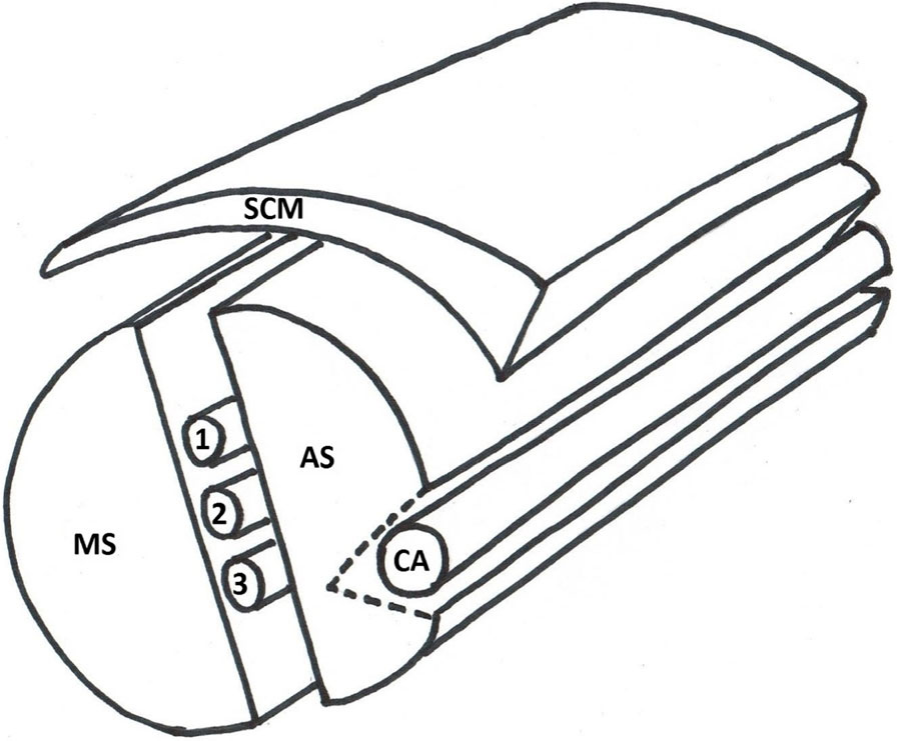
Cartoon representation of the interscalene brachial plexus model. SCM, sternocleidomastoid muscle; AS, anterior scalene muscle; MS, middle scalene muscle; CA, carotid artery; GV, great vessel; the interscalene brachial plexus is shown between the AS and MS muscles; 1, C5 nerve root; 2, C6 nerve root; 3, C7 nerve root

Make a lengthwise incision approximately 2 cm deep on the outer surface of one of the pieces of meat. The pocket that is created by this incision will contain the wide straw representing the carotid artery (the internal jugular vein is not represented in this model). Inject gel into a wide straw and occlude both ends with chewing gum. Sprinkle meat glue in this pocket and place the wide straw into it, firmly pressing the edges of the incision together. (Fig. 6a). Affix the thin wedge-shaped slice of meat representing the sternocleidomastoid muscle on top of the model with meat glue (Fig. 6b). A cartoon representation of the completed model is shown in Fig. 7.

#### Serratus anterior plane model

**Fig. 8:**
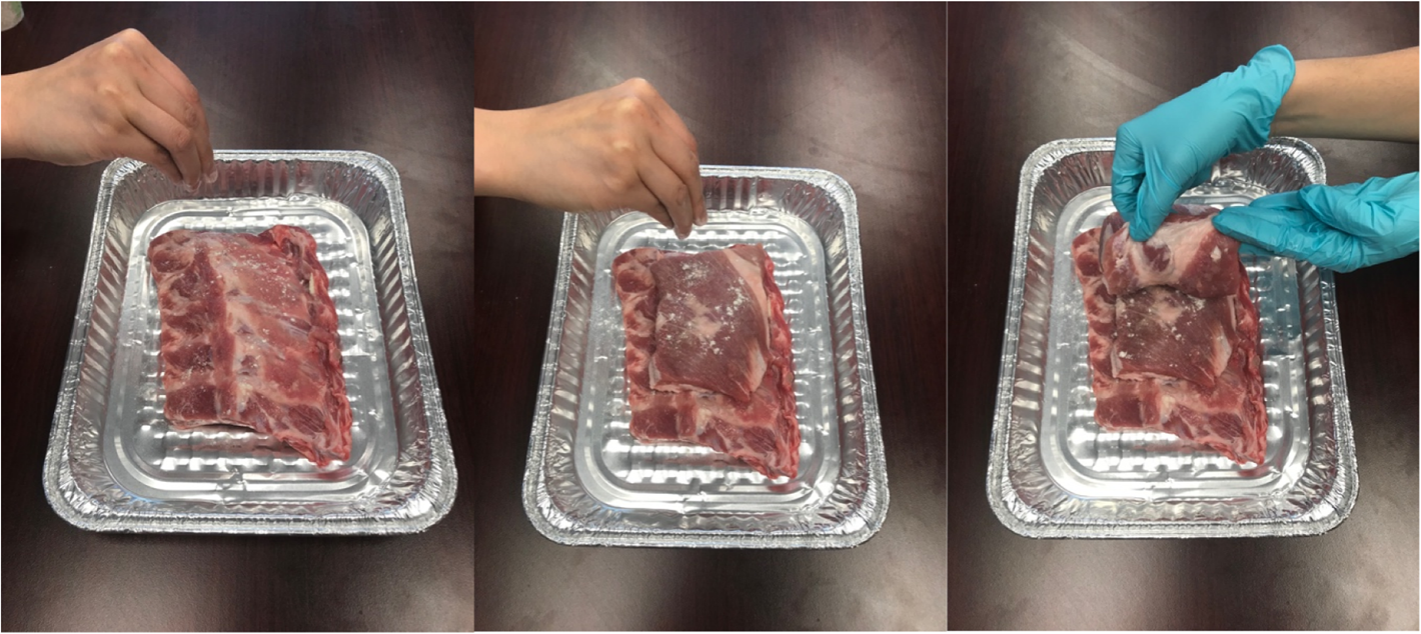
One row of pork rib and 2 slabs of meat placed on top of the pork rib with meat glue sprinkled between each layer

**Fig. 9:**
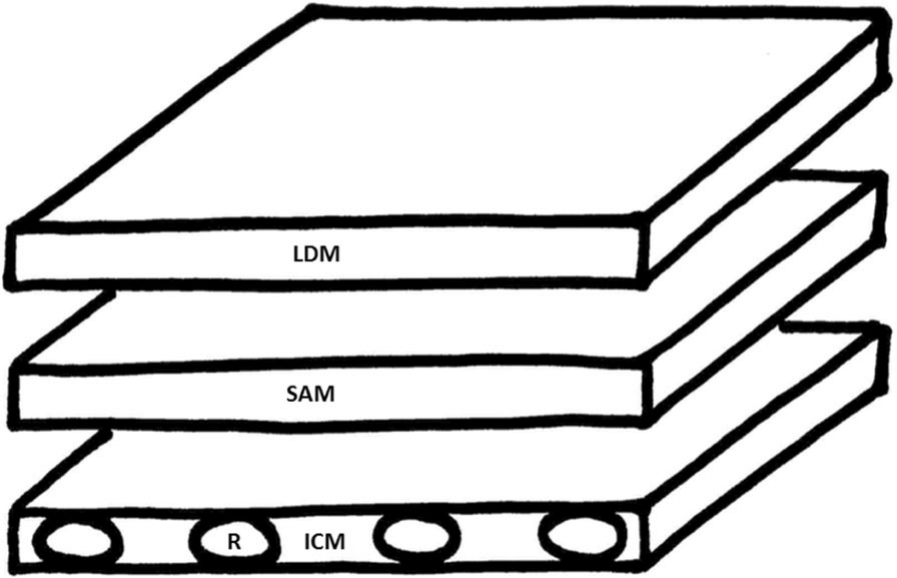
Cartoon image of the assembled serratus anterior plane model. LDM, latissimus dorsi muscle; SAM, serratus anterior muscle; ICM, intercostal muscle; R, rib

For this model, obtain one row of 5–6 attached pork ribs and two slabs of meat approximately 2 cm thick. Stack the layers of meat with the pork ribs at the bottom and the two other slabs on top and sprinkle meat glue between each layer (Fig. 8). The top layer of meat represents the latissimus dorsi muscle, the second layer represents the serratus anterior muscle and the row of attached pork ribs at the bottom represents ribs and intercostal muscles. The location of naturally occurring fascia apposition should occur between the top and second layer of meat just below. A cartoon representation of this model is shown in Fig. 9.

### UGRA model workshop

Forty board certified emergency medicine physicians participated in a workshop that consisted of a lecture on the serratus anterior plane block (SAPB) and fascia iliaca compartment block (FICB), followed by a one-hour hands-on session with both a live human model and ultrasound-guided injection of the UGRA meat model. Participants were emailed an anonymous online survey after the workshop that asked if they felt the SAPB and FICB meat model helped them practice the following four techniques: 1) visualizing the entire needle using the in-plane technique, 2) knowing where to deposit the anesthetic fluid, 3) hydrodissection, and 4) differentiating when the needle tip was in the muscle belly (incorrect place) versus the fascial plane (correct place).

## Results

### Peripheral nerve model

**Fig. 10:**
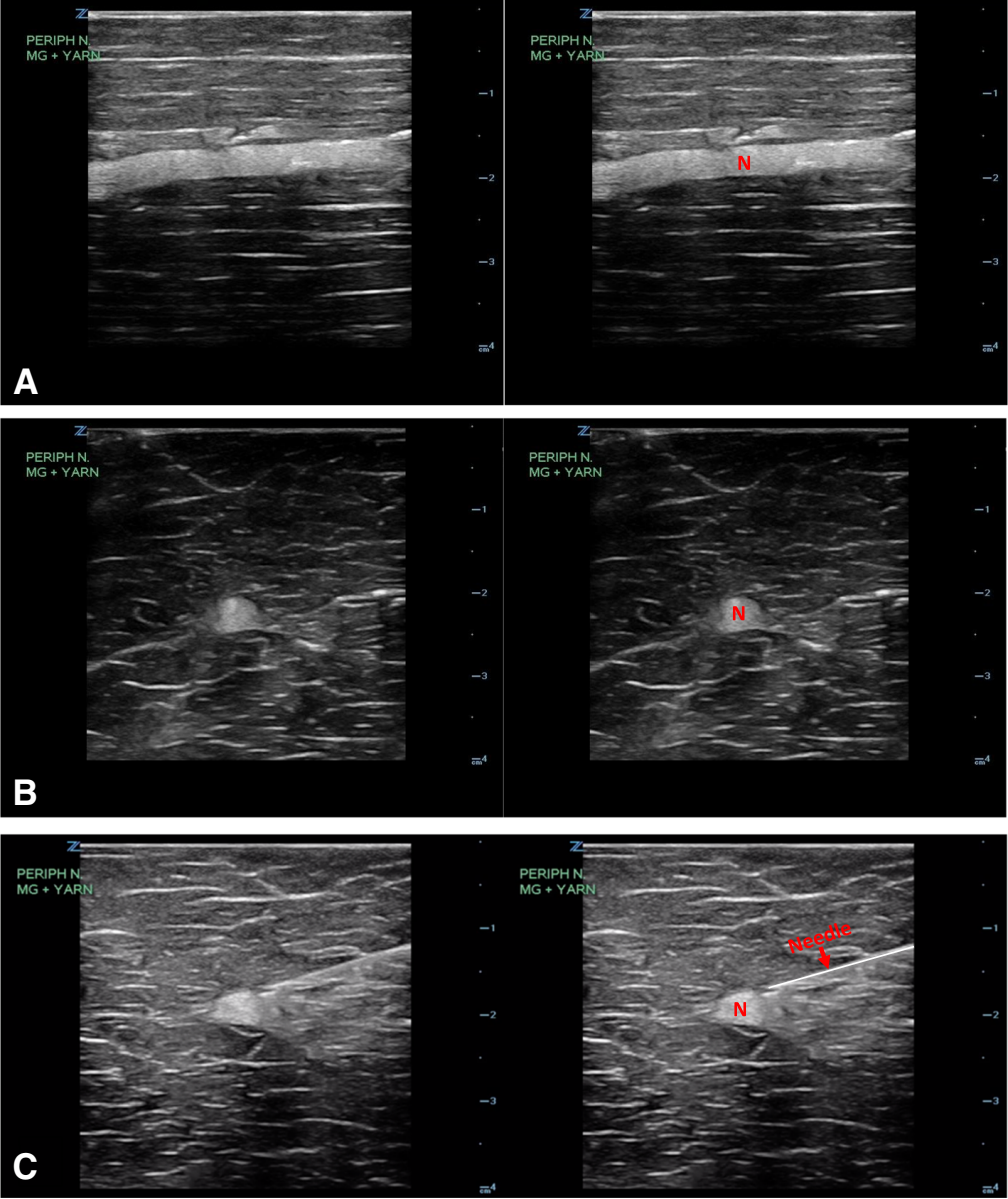
Ultrasound image of the peripheral nerve model in longitudinal (**a**) and transverse views (**b**). Ultrasound image of the transverse view with the needle placement (**c**). N, nerve

The sonographic appearance of the model is shown in Fig. 10 and fluid being injected into the peripheral nerve model under ultrasound guidance is demonstrated in Additional file 1: Video S1.

### Fascia Iliaca compartment model

**Fig. 11:**
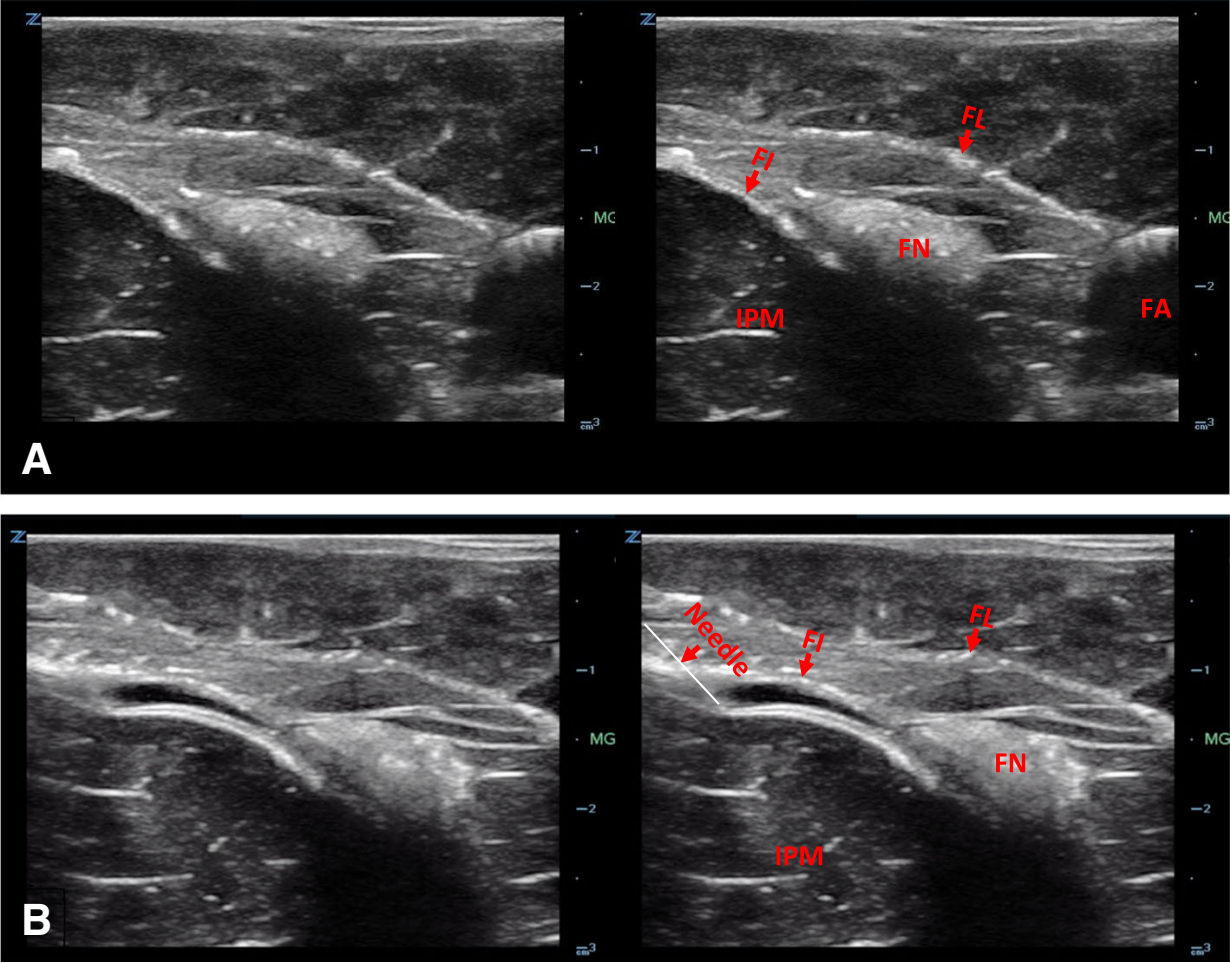
Ultrasound image of the fascia iliaca compartment model (**a**). Ultrasound image of the needle with hydrodissection of fascia iliaca plane (**b**). FN, femoral nerve; FA, femoral artery; IPM, iliopsoas muscle; FI, fascia iliaca; FL fascia lata

The sonographic appearance of the model is shown in Fig. 11 and fluid being injected into the femoral nerve model under ultrasound guidance is demonstrated in Additional file 2: Video S2.

### Interscalene brachial plexus model

**Fig. 12:**
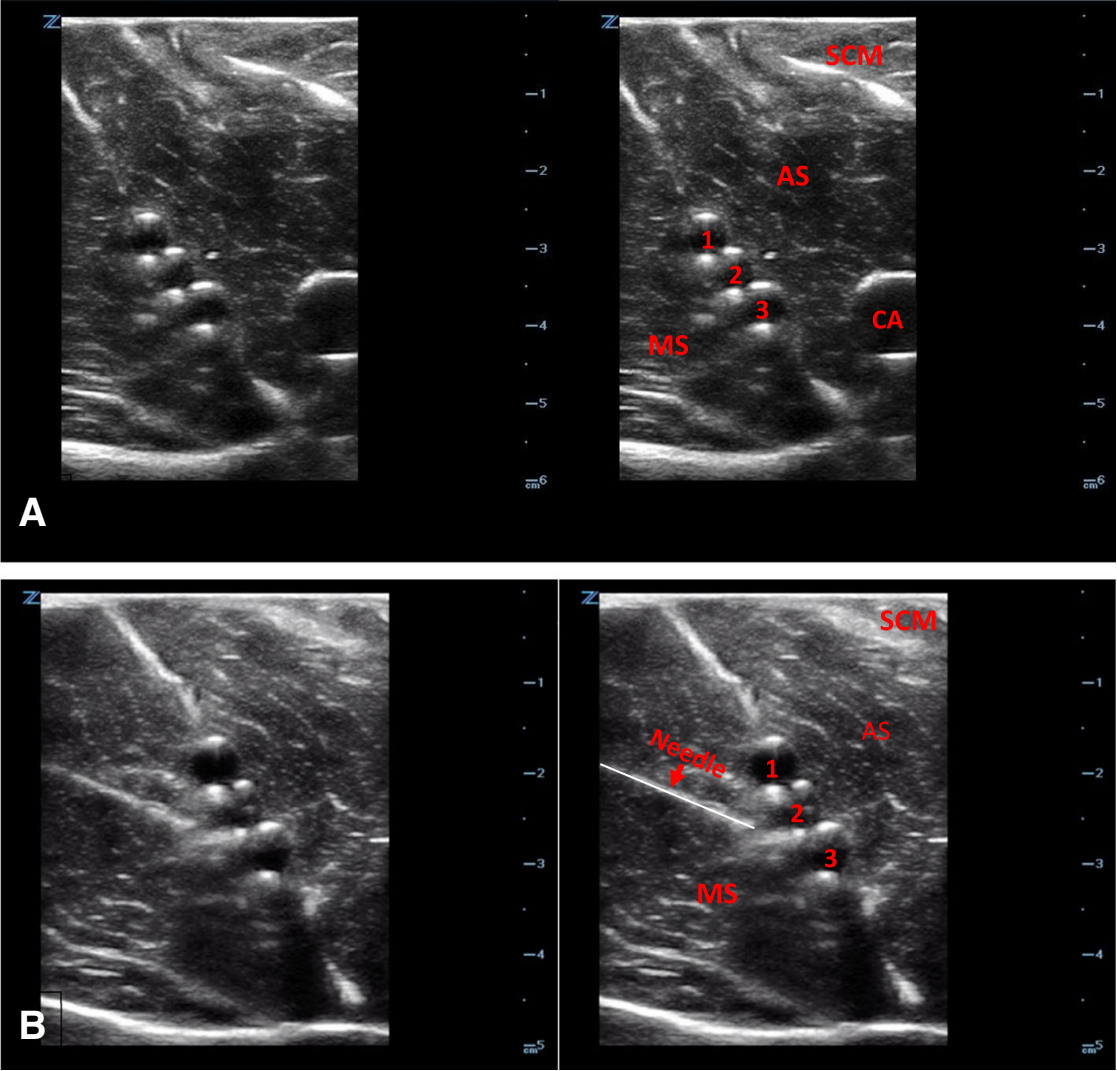
Ultrasound image of the interscalene brachial plexus model (**a**). Ultrasound image of needle placement (**b**). SCM, sternocleidomastoid muscle; AS, anterior scalene muscle; MS, middle scalene muscle; CA, carotid artery; IS, interscalene plexus; 1, C5 nerve root; 2, C6 nerve root; 3, C7 nerve root

The sonographic appearance of the interscalene brachial plexus model is shown in Fig. 12 and fluid being injected into the model under ultrasound guidance is demonstrated in Additional file 3: Video S3.

### Serratus anterior plane model

**Fig. 13:**
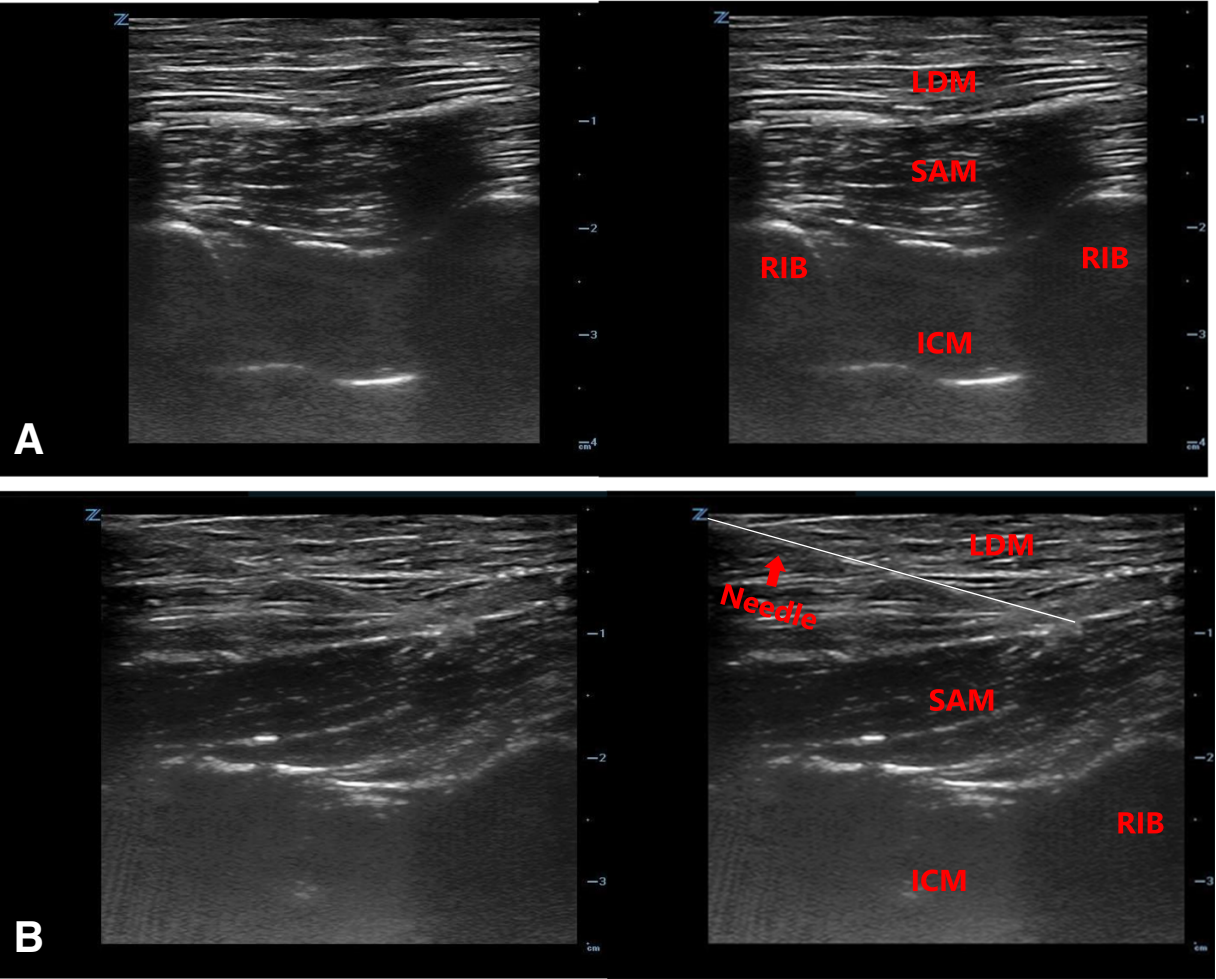
Ultrasound image of the serratus anterior plane model (**a**). Ultrasound image of needle placement (**b**)

The sonographic appearance of the serratus anterior plane model is shown in Fig. 13 and fluid injection into the model under ultrasound guidance is demonstrated in Additional file 4: Video S4.

### UGRA model workshop survey results

Out of forty attending physician participants, fourteen participants responded to an anonymous online survey after the workshop. All 14 respondents either agreed (6 out of 14, 43%) or strongly agreed (8 out of 14, 57%) that the SAPB meat model helped them practice each of the following four techniques: 1) visualizing the entire needle using the in-plane technique, 2) knowing where to deposit the anesthetic fluid, 3) hydrodissection, and 4) differentiating when the needle tip was incorrectly placed in the muscle belly (Additional file 5: Video S5) versus correctly placed in the fascial plane (Additional file 4: Video S4). When asked about the FICB meat model, all 14 respondents either agreed (7 out of 14, 50%) or strongly agreed (7 out of 14, 50%) that the FICB meat model helped them practice all four techniques.

## Discussion

It is essential that a UGRA meat model provide as close to a real-life training experience as possible. Our model accomplishes this with its realistic sonographic appearance of tissues, nerves, muscles, fascial planes, and blood vessels. There are several skills necessary in successfully performing UGRA. Performing in-plane technique and visualizing the needle tip on a meat model will mimic the appearance and feel of inserting the needle through muscle in a live UGRA. Another important skill involves placing the needle tip in the space either adjacent to the nerve or along a fascial plane. We found that when two layers of naturally occurring fascia were apposed together with meat glue, it will create a plane that can be hydrodissected mimicking the deposition of local anesthetic in real patients. Fluid injected into the space disrupts the fascia locally without disseminating to other areas along the fascial plane due to the binding ability of the meat glue. This helps differentiate correct injection into the fascial plane as opposed to incorrect injection into the muscle belly. The model can therefore be injected repeatedly by several learners along different sections of the fascia.

Our experience incorporating the UGRA meat models in teaching workshops has shown that learners are able to practice the aforementioned techniques using these models. A survey of emergency medicine attendings after use of the SAPB and FICB in a UGRA workshop found that 100% of respondents either agreed or strongly agreed that the models helped them practice in-plane technique, visualization of the needle tip, hydrodissection, and differentiation of the needle tip in the muscle belly versus the fascial plane.

The model is inexpensive and uses easily obtainable materials. The authors were able to create all four models described here for under 40 US Dollars. We found that the model can be frozen after initial use, thawed and used again for a second time. The ability to hydrodissect is retained even on second use. However, the model needs to be completely thawed or else the ice in the meat will cause acoustic shadowing that will diminish visibility.

The main limitation of these UGRA meat models is its perishability. We found that the models can be used no more than two separate workshops. However, each meat model can be injected repeatedly by several learners. These meat models also lack the external anatomy needed to help the trainee learn where to place the probe on a real person, therefore we recommend complementing these models with demonstration of external landmarks on a live model.

## Conclusion

We have developed inexpensive, easily reproducible, injectable models that can be customized to mimic the nerves, fascial planes, muscle, and blood vessels seen in a variety of nerve blocks. The use of meat glue allows trainees to learn ultrasound-guided nerve block techniques including the ability to hydrodissect fluid along a fascial plane. The authors are not aware of any other nerve block model construction techniques that offer the ability to visualize the injection of local anesthetic along a fascial plane. We believe that these models can help make UGRA education more effective, widespread and accessible for learners of all specialties. Further studies would need to be conducted to test the efficacy of these models when used as a medical training tool in teaching UGRA.

## Additional files

Additional file1: **Video S1.** Injection of water (simulating local anesthetic) around the peripheral nerve. (MP4 378 kb)

Additional file 2: **Video S2.** Hydrodissection of facial layers and the diffusion of injected water around the femoral nerve. (MP4 496 kb)

Additional file 3: **Video S3.** Injection and spread of water (simulating local anesthetic) around the interscalene nerve roots. (MP4 663 kb)

Additional file 4: **Video S4.** Correct needle placement showing the hydrodissection of serratus anterior facial plane. (MP4 403 kb)

Additional file 5: **Video S5.** Incorrect needle placement showing fluid deposition inside the muscle. (MP4 576 kb)

## References

[1] Beaudoin FL, Nagdev A, Merchant RC, Becker BM (2010). Ultrasound-guided femoral nerve blocks in elderly patients with hip fractures. Am J Emerg Med.

[2] Dickman E, Pushkar I, Likourezos A, Todd K, Hwang U, Akhter S, Morrison S (2016). Ultrasound-guided nerve blocks for intracapsular and extracapsular hip fractures. Am J Emerg Med.

[3] Luftig J, Mantuani D, Herring AA, Nagdev A (2017). Ultrasound-guided retroclavicular approach infraclavicular brachial plexus block for upper extremity emergency procedures. Am J Emerg Med.

[4] Nejati A, Teymourian H, Behrooz L, Mohseni G (2017). Pain management via ultrasound-guided nerve block in emergency department; a case series study. Emergency (Tehran, Iran).

[5] Fletcher AK, Rigby AS, Heyes FL (2003). Three-in-one femoral nerve block as analgesia for fractured neck of femur in the emergency department: a randomized, controlled trial. Ann Emerg Med.

[6] Christos SC, Chiampas G, Offman R, Rifenburg R (2010). Ultrasound-guided three-in-one nerve block for femur fractures. West J Emerg Med.

[7] Haines L, Dickman E, Ayvazyan S, Pearl M, Wu S, Rosenblum D, Likourezos A (2012). Ultrasound-guided fascia iliaca compartment block for hip fractures in the emergency department. J Emerg Med.

[8] Rahimzadeh P, Imani F, Sayarifard A, Sayarifard S, Faiz SH (2016). Ultrasound-guided fascia iliaca compartment block in orthopedic fractures: bupivacaine 0.2% or 0.3%?. Med J Islam Repub Iran.

[9] Raeyat Doost E, Heiran MM, Movahedi M, Mirafzal A (2017). Ultrasound-guided interscalene nerve block vs procedural sedation by propofol and fentanyl for anterior shoulder dislocations. Am J Emerg Med.

[10] Amini R, Patricia Javedani P, Amini A, Adhikari S (2016). Ultrasound-guided forearm nerve blocks: a novel application for pain control in adult patients with digit injuries. Case Rep Emerg Med.

[11] Frenkel O, Liebmann O, Fischer JW (2015). Ultrasound-guided forearm nerve blocks in kids: a novel method for pain control in the treatment of hand-injured pediatric patients in the emergency department. Pediatr Emerg Care.

[12] Milligan R, Houmes S, Goldberg LC, Nagdev A, Amini R (2017). Ultrasound-guided forearm nerve blocks in managing hand and finger injuries. Intern Emerg Med.

[13] Wroe P, O'Shea R, Johnson B, Hoffman R, Nagdev A (2016). Ultrasound-guided forearm nerve blocks for hand blast injuries: case series and multidisciplinary protocol. Am J Emerg Med.

[14] Chao SL, Chen KC, Lin LW, Wang TL, Chong CF (2013). Ultrasound phantoms made of gelatin covered with hydrocolloid skin dressing. J Emerg Med.

[15] Gibbs V (2015). The role of ultrasound simulators in education: an investigation into sonography student experiences and clinical mentor perceptions. Ultrasound (Leeds, England).

[16] Maul H, Scharf A, Baier P, Wustemann M, Gunter HH, Gebauer G, Sohn C (2004). Ultrasound simulators: experience with the SonoTrainer and comparative review of other training systems. Ultrasound Obstet Gynecol.

[17] Guidance for institutional review boards and clinical investigators. https://www.fda.gov/RegulatoryInformation/Guidances/ucm126420.htm.

